# Acupuncture-Analgesia-Mediated Alleviation of Central Sensitization

**DOI:** 10.1155/2019/6173412

**Published:** 2019-03-07

**Authors:** Hsiang-Chun Lai, Yi-Wen Lin, Ching-Liang Hsieh

**Affiliations:** ^1^Department of Chinese Medicine, China Medical University Hospital, Taichung 40447, Taiwan; ^2^Graduate Institute of Acupuncture Science, College of Chinese Medicine, China Medical University, Taichung 40402, Taiwan; ^3^Chinese Medicine Research Center, China Medical University, Taichung 40402, Taiwan; ^4^Research Center for Chinese Medicine and Acupuncture, China Medical University, Taichung 40402, Taiwan; ^5^Graduate Institute of Integrated Medicine, College of Chinese Medicine, China Medical University, Taichung 40402, Taiwan

## Abstract

Pain can trigger central amplification called central sensitization, which ultimately results in hyperalgesia and/or allodynia. Many reports have showed acupuncture has an analgesic effect. We searched the related article on PubMed database and Cochrane database to discover central sensitization pathway in acupuncture analgesia. We summarized that acupuncture enhances the descending inhibitory effect and modulates the feeling of pain, thus modifying central sensitization. The possible mechanisms underlying the analgesic effects of acupuncture include segmental inhibition and the activation of the endogenous opioid, adrenergic, 5-hydroxytryptamine, and N-methyl-D-aspartic acid, *α*-amino-3-hydroxy-5-methyl-4-isoxazolepropionic acid/kainate pathways. Moreover, acupuncture can locally reduce the levels of inflammatory mediators. In clinical settings, acupuncture can be used to treat headache, neuropathic pain, low back pain, osteoarthritis, and irritable bowel syndrome. These mechanisms of acupuncture analgesia may be involved in the alleviation of central sensitization.

## 1. Introduction

Pain is one of the most common clinical problems worldwide, and it adversely affects quality of life. The generation of pain results from tissue damage or similar pathophysiological causes. Signal transmission pathways of pain such as the spinothalamic pathway involve multiple gates and interfering effects to mislead the brain [1]. In its vicious cycle, a pain stimulus itself can trigger the central amplification of pain, called central sensitization, and ultimately cause hyperalgesia [2]. Pain management includes strategies such as pharmacotherapy, physical activity, social support, acupuncture, heating, rest, diets, or lifestyle changes [3]. Central sensitization is defined as “an amplification of neural signaling within the central nervous system (CNS) that elicits pain hypersensitivity” [4]. Therefore, central sensitization is due to a nociceptive input that results in a persistent increase in the excitability and synaptic effect of neurons in the nociceptive pathways of the CNS, and this phenomenon maintains a persistent state of heightened reactivity [4]. Central sensitization is due to an enhanced response of the CNS, which causes the development of hyperalgesia [5]. Altered membrane excitability, reduced inhibitory transmission, and increased synaptic efficacy contribute to the development of central sensitization. The lamina I and lamina V neurons of the spinal cord as well as the thalamus, amygdala, and anterior cingulate cortex are involved in central sensitization [6]. Therefore, central sensitization is due to a persistent state of high reactivity of nociceptive afferent neurons in the CNS. The pathological changes following tissue damage and nerve injury in the dorsal root ganglion (DRG) and dorsal horn of the spinal cord may create a state of chronic pain, and some of the mechanisms underlying this phenomenon are outlined as follows: (1) alteration of sodium and potassium ion channel expression in the DRG; (2) release of glutamate from the primary afferent neurons and increase in glutamate receptor function in the second-order neurons, as well as disinhibition of local inhibitory *γ*-aminobutyric acid (GABA)ergic and glycinergic interneurons in the dorsal horn of the spinal cord; and (3) release of cytokines and chemokines caused by the activation of spinal microglia and astrocytes [7]. Hyperalgesia (increased pain sensitivity) and allodynia (pain production induced by a nonnociceptive stimulation) are the two main characteristics of central sensitization [5]. Many clinical syndromes—such as rheumatoid arthritis, osteoarthritis, temporomandibular disorders, fibromyalgia, musculoskeletal disorders, tension-type headache, neuropathic pain, complex regional pain syndrome, and postsurgical pain—may contribute to central sensitization [4].

Acupuncture is a well-known treatment modality that originated in China. This procedure includes the insertion of needles into specific points of the body (called acupoints) to achieve therapeutic effects. According to the theory of traditional Chinese medicine (TCM), acupuncture modulates the flow of Qi and blood through the meridians and restores the balance of the five organs to maintain homeostasis [8]. To date, acupuncture is considered a valid treatment method for alleviating acute and chronic pain in clinical practice. Many studies have discussed the possible mechanism of pain reduction through acupuncture treatment.

In this article, we review the mechanisms of pain, the causes of central sensitization, and the mechanisms underlying acupuncture analgesia.

## 2. Methods

**Figure 1:**
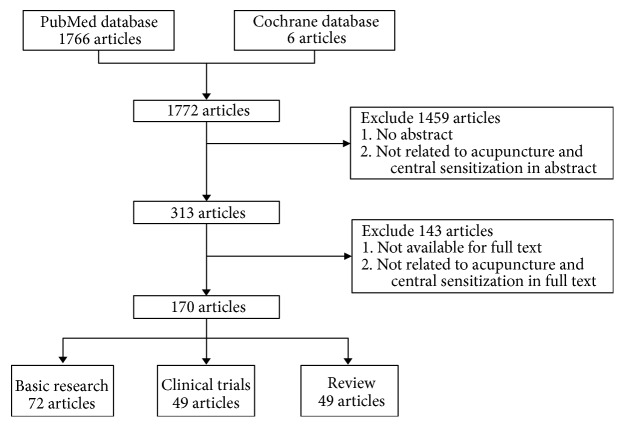
Flow chart of the search processes.

We searched the PubMed database and Cochrane database for studies published unlimited, beginning date to November 2017. The keywords included “acupuncture,” “pathophysiology,” “central sensitization,” “analgesia,” and “pain.” Language was limited to English and Chinese. The filter process was firstly by search engine of the website which yielded 1772 articles. We excluded 1459 articles due to no abstract or not related to acupuncture and central sensitization in abstract by the authors which yielded 313 articles. We excluded 143 articles due to no full text or not related to acupuncture and central sensitization in full text by the authors which yielded 170 articles. Therefore, the basic, clinical and review article were 72, 49 and 49, respectively, in type of article. The manuscript included basic and clinical studies related to central sensitization and acupuncture analgesia. Flow chart of the search processes was as shown in Figure 1.

### 2.1. Physiology of Pain

Somatic sensations are relays from the peripheral receptors to the brain cortex. Signals are transferred from distal nociceptors to the dorsal horn of the spinal cord (synapsing on second-order neurons) and through the brainstem to the ventral posterolateral nucleus of the thalamus. Finally, the signals are projected to the postcentral gyrus of the parietal cortex. The ascending transduction is called the lateral spinothalamic pathway or anterolateral system [9–11].

The descending modulatory pathway plays a crucial role in acupuncture analgesia. The pathway includes the cortex, ventrolateral (vl) periaqueductal gray (PAG, vlPAG) matter, rostral ventromedial medulla (RVM), locus coeruleus, raphe nucleus, and inhibitory synapses in the dorsal horn of the spinal cord [12].

### 2.2. Mechanism of Pain-Induced Central Sensitization

The sensory processing of pain is similar to a neural relay from the distinct pain-affected region of the body to the brain cortex. The upregulation or downregulation of each gate would interfere with the “feeling” of ordinary sensations. The threshold for activating primary afferent nociceptors is reduced under intense, repeated, or prolonged stimuli. The relatively low threshold of the nerve ending contributes to a relatively high frequency of firing for stimuli of all intensities. Central sensitization occurs with inflammatory mediators such as bradykinin, nerve-growth factor, some prostaglandins, leukotrienes, and nitric oxide [13]. After central sensitization, light stimuli can also produce pain; this condition is called allodynia.

In the dorsal horn of the spinal cord, the nociceptive nerve endings release glutamate and substance P, which act as postsynaptic* N*-methyl-D-aspartate (NMDA) and neurokinin-1 receptors during neural central sensitization. This mechanism prolongs the painful state [11]. In acute peripheral nerve injury, the loss of *γ*-aminobutyric acid (GABA)ergic interneurons reduces inhibitory control and increases the firing of the dorsal horn neurons [14]. Glia and immunocompetent cells in the dorsal horn secrete glutamate, cytokines, neurotrophins, nitric oxide, prostaglandins, and adenosine 5′-triphosphate. These elements can amplify the pain pathway [15]. Repeated stimuli were reported to increase the expression of ionizing calcium-binding adapter molecule 1 and cause microglial hypertrophy in an animal model [16].

Brain-derived neurotrophic factor (BDNF) has been identified as a critical regulator of neuronal development, synaptic transmission, and synaptic plasticity. BDNF can act on the dorsal horn neurons of the spinal cord and increase their excitability and spinal long-term potentiation, in addition to inducing inflammatory pain [17]. It can enhance synaptic facilitation and engage central sensitization-like mechanisms [18]. BDNF-containing neurons have been observed in PAG and RVM. Upregulation of tropomyosin receptor kinase B (TrkB), a BDNF receptor, after inflammation aggravates descending pain facilitation [19].

NMDA receptor (NMDAR) activation increases pain sensitivity of the spinal cord and brain [20, 21]. The main binding ligands are glutamate and glycine (or d-serine). Increased glutamate levels in the posterior cingulate gyrus, posterior insula, prefrontal cortex, and amygdala would cause dysregulation of pain processing in the central nervous system (CNS) [22]. Recently, BDNF was found to share the same downstream activator as NMDARs, TrkB signaling. The hypothesis was that both BDNF and other NMDAR ligands contribute to the hyperalgesia in the brain, and the maintenance of spinal long-term potentiation depends mainly on self-regenerating glial BDNF [23, 24].

In the CNS, enkephalins and endorphins bind to *μ*-opioid receptors, inhibit the release of substance P, and reduce the pain sensation. This effect was observed during mesencephalic reticular formation in the amygdala, PAG matter and RVM [25]. In the human brain, pain can cause structural changes in gray and white matter. These changes enable people to learn new skills and build behaviors. It also engenders the process of “learning chronic pain.” In a previous study, structural plasticity and glial hypertrophy were observed in the hippocampus and the subventricular zone [26].

**Figure 2:**
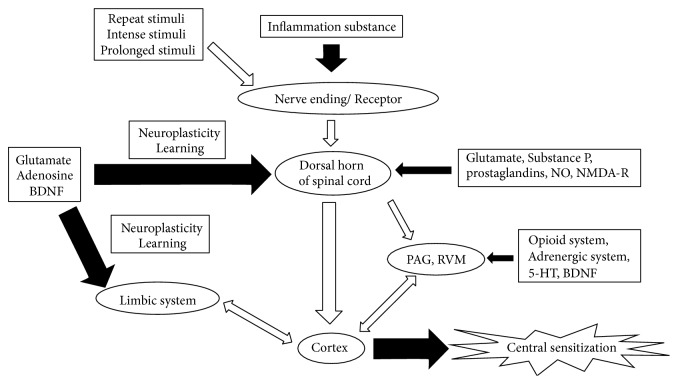
Mechanisms of pain-induced central sensitization. Pain transduction pathway (hollow arrows); upregulation of central sensitization (solid broad arrows); downregulation of central sensitization (solid thin arrows). 5-HT: 5-hydroxytryptamine; BDNF: brain-derived neurotrophic factor; NDMA: N-methyl-D-aspartic acid; NO: nitric oxide; PAG: periaqueductal gray; RVM: rostral ventromedial medulla.

Central sensitization can also be exaggerated and maintained because of cognition, attention, emotions, and motivation [27]. These factors can modify experiences of pain. A summary of the mechanisms of pain-induced central sensitization is presented in Figure 2.

### 2.3. Possible Mechanisms of Central Sensitization Reduction through Acupuncture Analgesia

#### 2.3.1. Segmental Inhibition or Gate Control Theory

A synapse in the dorsal horn of the spinal cord with a nociceptive nerve ending releases a neurotransmitter, which acts on postsynaptic receptors during neural central sensitization. The impulse of pain sensation is proportional to the number of sensitive loci and sensitized nociceptors involved. If these sensitized nociceptors send massive neural impulses to the spinal cord, it amplifies the central sensitization of exactly the same segments of the dorsal horn cells that govern a zone of pain referral [28, 29]. In the segment of needling, the pressure pain threshold increases, which indicates segmental inhibition in the spinal cord [30]. The segmental modulating mechanisms play a critical role in acupuncture analgesia which has been reported in a double-blind randomized controlled trial in patients with myofascial pain [31]

#### 2.3.2. Endogenous Opioid Pathway

The most well-known mechanism of acupuncture analgesia is the endogenous opioid pathway [32]. In experiments conducted on animal models, we have found that different frequencies of electroacupuncture (EA) caused different types of endogenous analgesia release; an EA treatment of 2 Hz accelerated the release of enkephalin, beta-endorphin, and endomorphin, and an EA treatment of 100 Hz increased the release of dynorphin [33–35]. Combining high and low frequencies can stimulate the release of four opioid peptides and provide the maximal therapeutic effect [34]. This analgesic process can be reversed by administering low doses of the opioid antagonist naloxone [36] and an antibody against encephalin or dynorphin [33]. We have concluded that the EA stimulation and opioid peptides share a common pathway in the CNS. Niddam et al. revealed that relieving pain through EA stimulation on a trigger point was mediated through the central pain modulation of the PAG in the brainstem [37]. Changes in PAG opioid activity were hypothesized to occur due to needling; needling may stimulate the nociceptive fibers, thus activating the enkephalinergic inhibitory dorsal horn interneurons [38].

The neuropeptide nociceptin/orphanin FQ (N/OFQ) is the endogenous agonist of the N/OFQ peptide receptor (NOP receptor). It was determined to have many physiological and pathological functions in pain regulation [39]. NOP receptors are found in the nucleus raphe magnus (NRM), dorsal raphe nucleus, and vlPAG [8, 40]. Fu et al. have reported that the levels of the precursor protein for N/OFQ increased and the N/OFQ immune reactivity decreased after peripheral inflammation in the superficial layers of the spinal dorsal horn [39, 41]. This process significantly increases after chronic inflammatory pain; however, it can be alleviated through EA treatment and warming moxibustion [42].

#### 2.3.3. Adrenergic Pathway

Norepinephrine is a potent inducer of analgesia in the spinal cord. In the descending pain modulation pathway, noradrenaline (norepinephrine)-containing neurons can be found in the raphe nuclei, locus coeruleus, PAG matter, and A1, A2, and A4-7 nuclei of the brainstem. These neurons project into the forebrain and pass through the dorsolateral tracts of the spinal cord [43]. Multiple animal studies have reported that acupuncture can reduce allodynia through the activation of an adrenergic mechanism [44–53]. Alpha2- and beta-adrenoceptors have been the most frequently reported receptors [46, 47, 51–53]. Chen et al. reported that alpha 2C receptors inhibit the release of opioids in the dorsal horn. Consequently, the activation of the adrenergic system can shut down the opioid system in the dorsal horn of the spinal cord segmentation [54].

#### 2.3.4. 5-Hydroxytryptamine Pathway

Serotoninergic neurons are found in the NRM, RVM, and trigeminal nucleus caudalis (TNC), and they project into the spinal cord [38]. In an inflammatory pain rat model, EA analgesia was mediated by a 5-hydroxytryptamine (5-HT) neurotransmitter, which binds to 5-HT1 and 5-HT3 receptors [55, 56]. This process can be reversed by 5-HT1 and 5-HT3 antagonists. Headache relief was considered to be primarily engendered by an increase in serotonin release in the medulla and TNC regions [57, 58]. Serotonin could inhibit inflammatory and neuropathic pain more effectively at 2–10 Hz than at 100 Hz [59]. Another study reported 5-HT1A and 5-HT3 receptors partially mediated the analgesic effects of EA at 2–10 Hz. By contrast, the 5-HT2 receptor was conversely involved in the nociceptive response at 100 Hz [60].

#### 2.3.5. NDMA/*α*-Amino-3-Hydroxy-5-Methyl-4-Isoxazolepropionic Acid/Kainate Pathway

Glutamate and aspartate are excitatory amino acids which bind to the NMDA/*α*-amino-3-hydroxy-5-methyl-4-isoxazolepropionic acid (AMPA)/kainate (KA) pathway and metabotropic receptors in the dorsal horn of spinal cord fiber terminals [61]. After prolonged and intense nociceptive impulse, substance P released in the dorsal horn increased the responsiveness of the NMDAR to glutamate and enhanced the spread of noxious input. This process also results in presynaptic modulation of astroglia which contributed to central pain sensitization [62].

Several animal studies have shown that EA treatment can attenuate the hyperalgesia of neuropathic pain through the downregulation of NMDAR phosphorylation at the spinal cord level [63–66]. EA decreases in the expression level of the NR-2B subunit of the NMDAR in the dorsal horn [51, 67–69]. Huang et al. revealed that combining low-dose ketamine, an NMDAR antagonist, with EA produced antiallodynic effects of a higher magnitude than did EA alone in a neuropathic pain model. This process could be reversed by naloxone, which indicates the possible interaction between the NMDA and endogenous opioid systems [64]. Gao et al. reported that EA at ST-36 causes the upregulation of NMDAR-mediated synaptic transmission and enhancement of gastric motility, which can alleviate irritable bowel symptoms [70].

#### 2.3.6. Local Inflammatory Environment

Local environment of active trigger zone is characterized by considerably higher levels of substance P, calcitonin gene-related peptide (CGRP), bradykinin (BK), 5-HT, norepinephrine, tumor necrosis factor-*α* (TNF-*α*), and interleukin-1*β* (IL-1*β*), compared with normal muscle tissue [71, 72]. These chemicals sensitize and activate muscle nociceptors, transfer impulse to the brain, and recruit spinal microglial cells for an inflammatory response in the microenvironment [73, 74]. The inflammation process causes neuronal hyperexcitability and amplifies nociception, resulting in chronic and neuropathic pain. Clinical improvement is accompanied by a reduction in the levels of inflammatory substances such as IL-1*β*, IL-8, IL-10, and TNF-*α* [8]. Administration of EA on GB-30 increases local C–X–C motif chemokine 10 (CXCL 10) production and activates the peripheral opioid peptide–mediated antinociception process [75], thus suggesting that acupuncture can cause an interaction between local opioid receptors and the mediators of anti-inflammatory responses. In addition, the possible pathways underlying the acupuncture-analgesia-mediated reduction in central sensitization are summarized in Table 1.

### 2.4. Acupuncture Analgesia Related to Central Sensitization

#### 2.4.1. Headache (Tension-Type Headache, Migraine, and Cluster Headache)

Headache is described using characteristics such as throbbing, dullness, tightness, or pressure in the head. It is primarily diagnosed as migraines, tension-type headaches, cluster headaches, or other secondary causes [76]. A headache is generally induced by tau band, stress, or the local release of inflammatory substances, and it is conducted via C fibers and A*δ* nociceptive neurons to the dorsal horn and trigeminal nucleus in the trigeminocervical complex, synapsing to the second-order neurons [77]. In the case of frequent and high intensity stimuli, these neurons are recruited via homosynaptic and heterosynaptic facilitation, which leads to the release of neuropeptides and neurotransmitters including NMDA, cyclooxygenase-2 (COX-2), nitric oxide, and fos [78–80]. A study on rats revealed that elevated levels of BDNF, a neuroplasticity mediator, in cerebrospinal fluid (CSF), result in synaptic plasticity [81]. The generated synaptic plasticity and accumulation of neurotransmitters, such as substance P and glutamate, can cause inefficiency diffused noxious inhibitory control and persistent sensitization, thus reducing pain thresholds and contributing to central sensitization of headache [80, 82].

*(1) Tension-Type Headache.* Patients with tension-type headache were found to have reduced pressure pain detection and tolerance thresholds in the temporal region compared with the controls [83]. The qualitative alteration in nociception was caused by central sensitization at the trigger point hyperalgesic zone and the level of the spinal dorsal horn and trigeminal nucleus [84, 85]. EA was demonstrated to block this pathway and inhibit neuroplasticity by reducing the BDNF level in a 29-participant human study [86].

*(2) Migraine.* The central sensitization pathophysiology of a migraine originates from persistent cutaneous hypersensitivity and general neuronal hyperexcitability and leads to RVM central sensitization [87]. Cutaneous allodynia is observed in migraine [88]. Boyer et al. demonstrated that repeated dural stimulation potentiates touch-induced fos expression in the trigeminal and spinal dorsal horns and causes diffuse noxious inhibitory control impairment and widespread, trigeminal, and spinal central sensitization [82].

In a randomized controlled trial involving 275 patients with migraine, EA on GB-40 was found to cause a significant difference in the visual analgesic scale scores of the EA and control groups. This effect of EA was accompanied by elevated 5-HT levels in the EA group [89]. EA also induced upregulation of cannabinoid receptor type 1 (CB1), resulting in the inhibition of the inflammatory effects of IL-1*β*, COX-2, Prostaglandin E2, and CGRP, in a migraine rat model [90].

*(3) Cluster Headache.* Cluster headache is a relatively rare type of primary headache but probably the most disabling and painful type [91]. The possible pathophysiology of cluster headache is associated with central sensitization of the brainstem and, possibly, thalamic neurons [92]. Fernández et al. observed widespread pressure pain hypersensitivity in patients with cluster headache, compared with healthy controls [93]. In addition, cluster headache patients were observed having decreasing plasma methionine-enkephalin levels [94]. However, lower CSF met-enkephalin levels in patients with cluster headache can be increased by manual acupuncture or EA [95].

In summary, acupuncture treats headache through the inhibition of neuropeptide (substance P), neurotransmitters (glutamate), and BDNF, as well as the release of opioid substances.

#### 2.4.2. Neuropathic Pain

Allodynia and hyperalgesia are common symptoms in patients with neuropathic pain. The prevalence of chronic pain with neuropathic characteristics was reported to range from 3% to 17% [96].

The origin of neuropathic pain is the input of terminal C fibers and A*β* fibers, which transfer signals to second-order projection neurons in the spinal cord. C fiber overactivation by capsaicin amplification in the spinal cord signaling systems causes central sensitization [97]. Landerholm et al. found that the modality of the evoked sensation changed from dynamic mechanical allodynia to dynamic mechanical dysesthesia after gradually increasing the compression block of A*β* input. This finding indicates that A*β* input is crucial to the presence of allodynia and is part of the spectrum of dysesthesia [98]. After nerve injury, second-order neurons are excited by increased input from the healthy area and nonnoxious input from damaged or undamaged A*β* fibers which cause central sensitization. Both types of repetitive stimuli may cause pain. Acupuncture attenuates nociceptive behavior and reduces mechanical allodynia by activating the components of the local molecular signaling pathway, mainly extracellular-signal-regulated kinase (ERK) [99]. This effect explains why acupuncture can be widely used for treating neuropathic pain. Additionally, a change in the balance of descending inhibitory and activating pathways from the brain to the spinal cord modulates dorsal horn neuronal activity and causes analgesic effect through central sensitization [97].

From a molecular viewpoint, allodynia has been determined to be accompanied by elevation of neuropeptides such as CGRP, substance P, and the neurotrophin BDNF in A*β* fibers [100, 101]. Animal studies have also revealed that acupuncture causes a reduction in glycine inhibition [102] and an increase in the activity of the neurotrophin BDNF (causing neuron plasticity) [101], NMDA, AMPA, and metabotropic glutamate receptors in the postsynaptic neurons [103]. Acupuncture increases the levels of these neuropeptides, including opioids, serotonin, norepinephrine, and amino acids and reduces the levels of the local inflammatory cytokines and the expression of their receptors [59, 63]. In a neuropathic pain rat model, repeated EA had a time-dependent cumulative analgesic effect; this might be associated with its modulatory effects on NK cells, as well as on splenic IL-2, *β* -Endorphin (*β*-EP), and plasma IL-2, IL-1*β*, interferon gamma (IFN-*γ*), and transforming growth factor beta (TGF-*β*) levels [104]. Many reports found that acupuncture can relieve neuropathic pain induced by postherpetic, multiple sclerosis, cancer and anticancer treatment, etc. in humans [105–108].

*(1) Postherpetic Neuralgia.* Postherpetic neuralgia (PHN) pain is characterized by a deep, burning, and throbbing ache as well as a sharp, stabbing, shooting, lancinating pain [109]. The prevalence of PHN-associated neuropathic pain was reported to be 3.9–42.0 per 100,000 person-years [96]. Allodynia was observed in at least 70% of patients with PHN. The identified risk factors for PHN include advancing age, high levels of acute pain, severe rashes, prodromal pain, ophthalmic location, and possibly female sex [110]. Although PHN is a vexing symptom, only a few systemic studies have been conducted on the use of acupuncture for the treatment of this condition.

In animal studies, EA was shown to alleviate PHN through recovering transient receptor potential vanilloid type-1 (TRPV1)-positive sensory neurons [111] and reducing cerebral TRPV-4 expression [112]. Regarding human studies, a single-blind randomized controlled study of acupuncture compared with placebo was conducted on 62 patients with PHN; the results suggested that acupuncture is effective in treating PHN [108]. Lui et al. recommended methods for selecting Ashi points and Huatuojiaji points to treat PHN [113].

*(2) Trigeminal Neuralgia.* The prevalence of neuropathic pain associated with trigeminal neuralgia (TN) was revealed to be 12.6–28.9 per 100,000 person-years [96]. Patients experienced intense paroxysmal pain and described it as being similar to an electric shock sensation (“painful flash”) that lasts approximately 1 second and may recur within minutes. This pain is always unilateral and typically limited to the second or third branch of the trigeminal nerve. A trigger zone may occasionally exist which could cause an episode of pain after touching or stretching [114]. Acupuncture can significantly increase the levels of plasma ß-endorphin and ß-lipotropin in patients with TN [115].

*(3) Diabetic Peripheral Neuropathy.* The prevalence of diabetic peripheral neuropathy (DPN)-associated neuropathic pain was shown to be 15.3–72.3 per 100,000 person-years [96]. Diabetic neuropathy affects up to 50% of patients with diabetes for 25 years, and painful DPN occurs in 26.4% of all people with diabetes [116]. The degree of pain ranges from mild dysesthesias to severe unremitting pain that considerably hinders the patients' lives [117]. Studies on DPN have reported increased glutamate release from the primary afferent neurons and reduced function of the presynaptic GABA_B_ receptors in the dorsal horn of the spinal cord [118, 119]. Spinal NMDAR overexpression frequently excites the postsynaptic lamina II neurons. Moreover, augmented NMDA expression and glutamate release might contribute to spinal cord hyperactivity [120]. The activation of GABA_B_ receptors, reduction in NMDAR expression in the spinal cord dorsal horn [121], and increase in norepinephrine and 5-HT levels in the spinal cord as well as RVM neurons were noted in DPN rats [122]. These facilitation pathways account for central sensitization in diabetic neuropathy. Consequently, acupuncture provides an effective treatment [123].

Several one-arm studies have reported acupuncture to be a safe and effective therapy for painful diabetic neuropathy [124–126]. The mechanisms underlying the analgesic effects of acupuncture might be mediated by the inhibition of the NF-*κ*B signaling pathway in primary sensory neurons and substance P, as seen in rat models [127, 128]. At the spinal cord level, EA can increase the glutamic acid decarboxylase-67 (GAD-67) level, reduce the TRPV-1 level, and modulate the nerve-growth factor level in rat models [128].

In summary, the mechanisms though which acupuncture alleviates neuralgia mainly involve the enhancement of the descending inhibition pathway, including the release of opioids and the inhibition of NMDA. In addition, the inhibition of local inflammation and TRPV1 receptors play a role in the alleviation of neuralgia.

*(4) Central Poststroke Pain.* Patients with central poststroke pain (CPSP) experience a continuous or paroxysmal pain, which was described in a previous study as burning, aching, pricking, squeezing, or throbbing either in isolation or in various combinations of the aforementioned descriptions [129]. The pain becomes severe after any stimuli, such as movement, touch, temperature, or stress. Allodynia, dysesthesia, and hyperalgesia affect 33%–86% of patients with CPSP [130]. CPSP mostly develops on the contralateral side to the stroke within 6 months of stroke onset, and its incidence decreases with time [131]. The most common pain is poststroke shoulder pain, which occurs on the affected side after 2 to 3 months [132, 133].

The mechanism of CPSP has been attributed to disinhibition theory, implying the imbalance of stimuli and contribution to central sensitization [134], chronic nociceptive [135, 136], or neuropathic pain [137–139]. Lateral thalamus dysfunction frees the medial thalamus. Then, the spinothalamocortical pathway becomes prominently overactive in the lateral thalamus and causes allodynia or dysaesthesia [129]. Localized neurogenic inflammation induces the initial phases of complex regional pain syndrome, causing repeated stimulation of the C fibers and increased medullary excitability (central sensitization) [140]. Frequent stimuli contribute to the CNS plasticity and consolidation of allodynia/dysaesthesia [141]. In a functional magnetic resonance imaging survey, acupuncture stimulation was shown to activate the limbic system, including the parahippocampal gyrus and anterior cingulate cortex, thus causing a central analgesic effect. This result may provide a clue regarding the analgesic mechanism of acupuncture [142]. Salom-Moreno et al. demonstrated that pain thresholds increased bilaterally in patients receiving needling, compared with those who did not [143].

#### 2.4.3. Low Back Pain (LBP)

Low back pain has etiology including muscles, nerves, and bones of the back. Patients would suffer from pain, limited physical activity, and sleep interference. Analgesics can provide temporary pain relief but have intolerable adverse effects for some patients. Thus, alternative treatments such as acupuncture [144], EA, transcutaneous electrical nerve stimulation (TENS), spine manipulation, and exercise therapy were options for these patients.

Patients with LBP have lower pressure pain threshold than healthy individuals suggesting sensitization of the central nervous system [145]. This effect might be due to segmental hyperresponsiveness, hyperalgesia (thermal stimuli), and enhanced temporal summation compared to healthy group which altered central nociceptive processing and caused chronic pain status [146]. Lam demonstrates in a systematic review that acupuncture has benefit in self-reported pain and functional limitations [147]. Another meta-analysis of randomized controlled trials suggests heat-sensitive moxibustion and acupuncture can improve lumbar disc herniation [148]. This result was correlated to widespread oscillatory changes in electroencephalography [149].

#### 2.4.4. Osteoarthritis Joint Pain

Osteoarthritis can be observed as breakdown of joint cartilage and underlying bone causing joint pain, swelling, decreased range of motion, and daily activities limitations. Treatment includes lifestyle change, medication, surgery, and alternative treatments. Acupuncture served as an option to prevent the degeneration of cartilage [150] and pain relief [151]. The central sensitization mechanism of osteoarthritis includes disturbance in nociceptive processes, local and widespread hyperalgesia, enhanced temporal or spatial summation, dysfunction of opioid and nonopioid system, and disturbance of proinflammatory cytokines neuropeptide [152].

EA attenuates the osteoarthritic pain by opioidergic receptors, 5-HT1, 5-HT3 receptor and muscarinic cholinergic receptors [55, 153, 154]. EA triggers chemokine CXCL10 to increase opioid-containing macrophages and reduce inflammatory pain [75]. Moxibustion relieves osteoarthritic pain also mediated by endogenous opioids pathways [155].

#### 2.4.5. Irritable Bowel Syndrome (IBS)

IBS patients suffered from recurrent abdominal pain and changes in the pattern of bowel movements without organic disease. It might be triggered by infection, intestinal bacterial overgrowth, stress, food sensitivity, gastrointestinal motility, visceral hypersensitivity, and brain-gut axis problems. Studies revealed IBS patients were more sensitive to pain which related to central dysfunction of viscerosomatic pathway [156, 157].

Acupuncture relieves IBS symptoms in many reports [158–161]. This effect was mediated by regulation of visceral hypersensitivity [162, 163]. EA decreases substance P in colon of rats [164, 165] and modulates brain-gut axis through decreasing 5-HT, CGRP, CRF, somatostatin, and NMDAR-1 and increasing NPY [166–170].

## 3. Conclusion

Acupuncture is a process that entails inserting needles into acupoints, triggering large myelinated A*β*- and A*δ*-fibers and transducing a neural signal to postcentral gyrus of the parietal cortex. The descending pathway passes through the raphe nucleus, locus coeruleus, PAG, prefrontal cortex, insula, cingulate cortex, caudate nucleus, amygdala, and inhibitory synapse in the dorsal horn. The descending pathway modulates the feeling of pain, which interferes with the central sensitization process.

The possible mechanisms through which acupuncture reduces central sensitization include segmental inhibition, the release of the endogenous opioid, adrenergic and 5-HT, and NMDA/AMPA/KA pathways. The local effects of acupuncture involve the reduction in the levels of inflammatory mediators such as substance P, IL-1*β*, IL-8, IL-10, and TNF-*α*. In summary, acupuncture acts through multiple pathways to produce analgesic effects and reduce central sensitization. Therefore, acupuncture is beneficial for the treatment of headache, neuropathic pain, low back pain, osteoarthritis, and irritable bowel syndrome.

## Figures and Tables

**Table 1:** Possible pathways through which acupuncture analgesia alleviates central sensitization.

Mechanism	Related part of neuron/nucleus
(1) Segmental inhibition	dorsal horn
(2) Endogenous opioid pathway	dorsal horn, PAG, NRM, dorsal raphe nucleus
(3) Adrenergic pathway	dorsal horn, PAG, raphe nuclei, locus coeruleus and brainstem (A1, A2, A4-7 nuclei), forebrain
(4) 5-Hydroxytryptamine pathway	NRM, RVM, trigeminal nucleus caudalis
(5) NDMA pathway	dorsal horn
(6) Local inflammatory environment	nerve ending, dorsal horn

NMDA: N-methyl-D-aspartic acid; PAG: periaqueductal gray; NRM: nucleus raphe magnus; RVM: rostral ventromedial medulla.
